# The Effects of *Poncirus fructus* on Insulin Resistance and the Macrophage-Mediated Inflammatory Response in High Fat Diet-Induced Obese Mice

**DOI:** 10.3390/ijms20122858

**Published:** 2019-06-12

**Authors:** Mia Kim, Mi Hyeon Seol, Byung-Cheol Lee

**Affiliations:** 1 Department of Cardiovascular and Neurologic Disease (Stroke Center), College of Korean Medicine, Kyung Hee University, 23 Kyungheedae-ro, Dongdaemun, Seoul 02447, Korea; hyuntemia@hanmail.net; 2 Department of Clinical Korean Medicine, Graduate School, Kyung Hee University, 26 Kyungheedae-ro, Dongdaemun-gu, Seoul 02447, Korea; smhj9658@daum.net

**Keywords:** *Poncirus fructus*, insulin resistance, inflammation, adipose tissue macrophages, Kupffer cells

## Abstract

Obesity is a chronic low-grade inflammatory condition in which hypertrophied adipocytes and adipose tissue immune cells, mainly macrophages, contribute to increased circulating levels of proinflammatory cytokines. Obesity-associated chronic low-grade systemic inflammation is considered a focal point and a therapeutic target in insulin resistance and metabolic diseases. We evaluate the effect of *Poncirus fructus* (PF) on insulin resistance and its mechanism based on inflammatory responses in high-fat diet (HFD)-induced obese mice. Mice were fed an HFD to induce obesity and then administered PF. Body weight, epididymal fat and liver weight, glucose, lipid, insulin, and histologic characteristics were evaluated to determine the effect of PF on insulin resistance by analyzing the proportion of macrophages in epididymal fat and liver and measured inflammatory gene expression. PF administration significantly decreased the fasting and postprandial glucose, fasting insulin, HOMA-IR, total-cholesterol, triglycerides, and low-density lipoprotein cholesterol levels. The epididymal fat tissue and liver showed a significant decrease of fat accumulation in histological analysis. PF significantly reduced the number of adipose tissue macrophages (ATMs), F4/80^+^ Kupffer cells, and CD68^+^ Kupffer cells, increased the proportion of M2 phenotype macrophages, and decreased the gene expression of inflammatory cytokines. These results suggest that PF could be used to improve insulin resistance through modulation of macrophage-mediated inflammation and enhance glucose and lipid metabolism.

## 1. Introduction

Obesity, an excess accumulation of body fat resulting from energy imbalance, is a chronic state of low-grade inflammation [1] that is well documented as a contributing factor in the development of diabetes, cardiovascular disease, and non-alcoholic fatty liver disease [2,3]. Adipose tissue, in addition to its ability to store lipids, is now recognized as an endocrine organ that produces various types of molecules to induce metabolic changes [4]. Adipocytes are hypertrophied in response to over-nutrition, increased adipose tissue ER stress, and hypoxia. Adipose tissue stress induces chemokine and pro-inflammatory cytokine production and leads to the progressive infiltration of immune cells [5,6]. Among these immune cells, macrophages play a critical role in obesity-associated adipose tissue inflammation [7]. Macrophage-derived TNF-α increases the release of free fatty acids via lipolysis, leading to ectopic fat deposition in liver and skeletal muscle [8,9]. Elevated circulating free fatty acids serve as ligands for the TLR4 complex [10], and the activated TLR4/NF-kB pathway induces cytokines, such as TNF-α and IL-6, which cause chronic inflammation and inhibit insulin signaling pathways [11]. Therefore, modulating the inflammation of adipose tissue should be a key treatment for obesity associated metabolic complications [12].

*Poncirus fructus (PF)*, the dried immature fruits of *Poncirus trifoliata* Rafinesque, has been used in the treatment of dyspepsia, as a prokinetic [13], and for improving blood circulation [14]. Many pharmacological studies have shown that *Poncirus* exerts anti-inflammatory [15], antioxidant [16], and lipid-lowering activities [17] along with gastroprotective effects [18] both in vitro and in vivo. Thus, in this study, we investigated the effects of PF on macrophage-mediated inflammatory responses and insulin resistance in high-fat diet fed C57BL/6 mice.

## 2. Results

### 2.1. Effects of PF on Body Weight and Epididymal Fat and Liver Weight Changes

**Figure 1:**
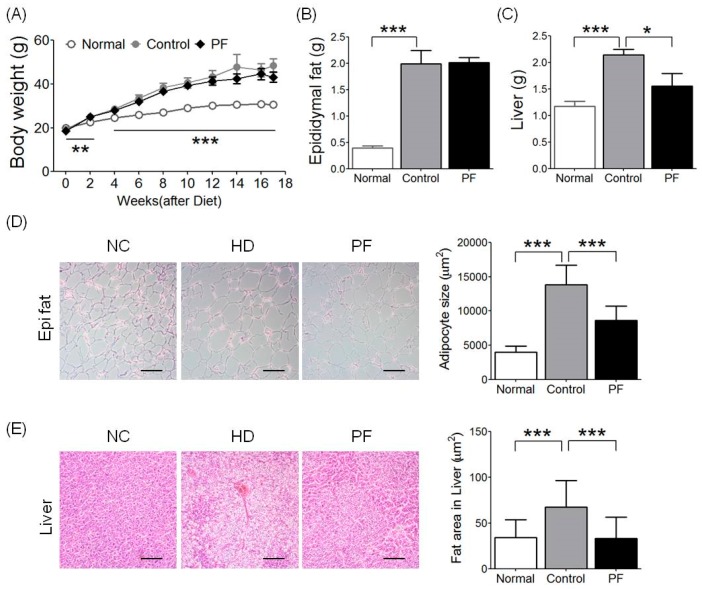
Effects of *Poncirus fructus* (PF) on (**A**) body weight, (**B**) epididymal fat, (**C**) liver weight, and (**D**,**E**) histological changes in epididymal fat and liver. Representative histological images were assessed by hematoxylin and eosin (H&E) staining, scale bar indicates 100 μm. (**D**) Adipocyte size in epi fat and (**E**) adipocyte size and fat area in liver. *n* = 6 in each group. Data shown are the mean ± standard error of the mean (SEM). * *p* < 0.05, ** *p* < 0.01, *** *p* < 0.001, compared with high-fat diet (HFD).

Higher body weight gain was observed in the high-fat diet (HFD) and PF groups than in the normal chow (NC) group. While body weight of the PF group was relatively low compared to the HFD group (43.14 ± 2.34 g vs. 48.42 ± 3.10 g, respectively), no significant difference in body weight gain was seen (Figure 1A). The HFD group showed a meaningfully higher epididymal fat pad weight compared to the NC group (*p* < 0.001), but the PF group did not show significant reductions in epididymal fat pad weight compared to the HFD group (2.01 ± 0.10 g vs. 2.00 ± 0.25 g, respectively, Figure 1B). The gap in liver weight was significant, with the HFD group being heavier than the NC group (*p* < 0.001). The PF group showed an even lower liver weight than the HFD group (1.56 ± 0.23 g vs. 2.15 ± 0.10 g, respectively; *p* < 0.05, Figure 1C).

### 2.2. Effects of PF on Fat Accumulation on Epididymal Fat and Liver

In the case of adipocyte size, the HFD group was significantly larger than the NC group (*p* < 0.001), and the PF group was significantly lower compared to that of the HFD group in epididymal fat tissue (8606.53 ± 2130.54 μm^2^ vs. 13,832.32 ± 2875.07 μm^2^, respectively; *p* < 0.001, Figure 1D). Liver fat area was also significantly higher in the HFD group compared to the NC group (*p* < 0.001), and liver fat area in the PF group was significantly lower than in the HFD group (33.05 ± 23.55 μm^2^ vs. 67.51 ± 29.20 μm^2^, respectively; *p* < 0.001, Figure 1E).

### 2.3. Effects of PF on Insulin Resistance and Glucose Tolerance Test

**Figure 2:**
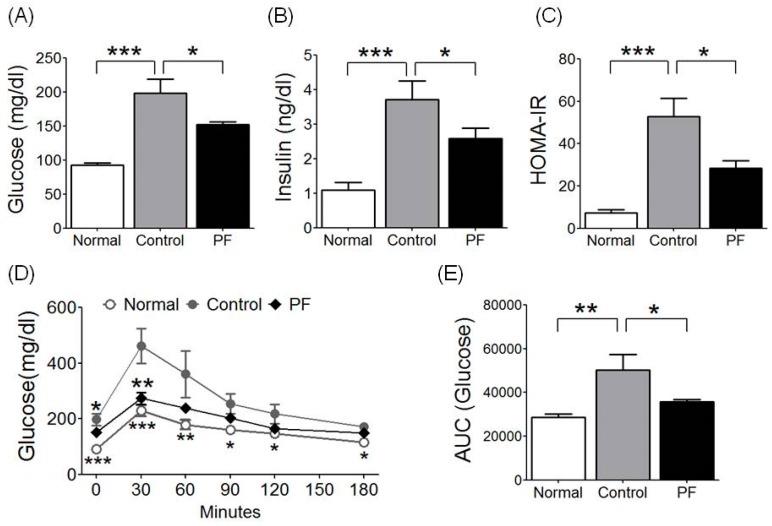
Effects of PF on (**A**) fasting glucose, (**B**) fasting insulin, (**C**) homeostatic model assessment for insulin resistance (HOMA-IR), and (**D**,**E**) oral glucose tolerance test (OGTT) and area under curve (AUC), respectively. *n* = 6 in each group. Data shown are the mean ± SEM. * *p* < 0.05, ** *p* < 0.01, *** *p* < 0.001, compared with HFD.

Insulin resistance was identified through measuring homeostatic model assessment for insulin resistance (HOMA-IR). HOMA-IR was significantly higher in the HFD group compared with the NC group (52.73 ± 8.74 vs. 7.28 ± 1.63, respectively; *p* < 0.001), and it was significantly lower in the PF group compared with the HFD group (28.31 ± 3.63 vs. 52.73 ± 8.74, respectively; *p* < 0.05, Figure 2C). Fasting glucose level was significantly higher in the HFD group compared with the NC group (198.33 ± 20.69 vs. 92.50 ± 3.07, respectively; *p* < 0.001), but it was significantly decreased by PF treatment (152.00 ± 4.12, *p* < 0.05, Figure 2A). Fasting insulin level also was significantly higher in the HFD group compared with the NC group (3.732 ± 0.54 vs. 1.09 ± 0.22, respectively; *p* < 0.001), and it was significantly lower in the PF group compared with the HFD group (2.58 ± 0.30 vs. 3.732 ± 0.54, respectively; *p* < 0.05, Figure 2B). From the oral glucose tolerance test (OGTT), glucose levels of all groups were highest at 30 min and then gradually decreased. The HFD group had a significantly higher blood glucose level than the NC group at every time point, while the PF group had significantly lower blood glucose levels than the HFD group at 0 min and 30 min (0 min, *p* < 0.05; 30 min, *p* < 0.01, Figure 2D). The HFD group had a significantly higher glucose area under curve (AUC) compared to the NC group (*p* < 0.01) and the PF group had a significantly lower AUC compared to the HFD group (35,724.00 ± 985.65 vs. 50,292.50 ± 7196.67, respectively; *p* < 0.05, Figure 2E).

### 2.4. Effects of PF on Lipid Metabolism and Fat Tolerance Test

**Figure 3:**
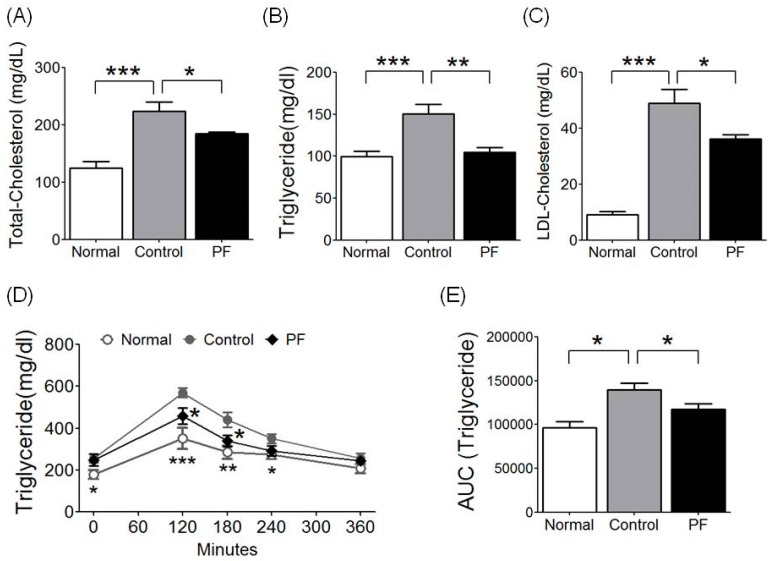
Effects of PF on (**A**) serum total cholesterol, (**B**) triglyceride, (**C**) low-density lipoprotein (LDL) cholesterol, and (**D**,**E**) oral fat tolerance test (OFTT) and area under curve (AUC), respectively. *n* = 6 in each group. Data shown are the mean ± SEM. * *p* < 0.05, ** *p* < 0.01, *** *p* < 0.001, compared with HFD.

For the evaluation of lipid profiles, total cholesterol (TC), triglyceride (TG), and low-density lipoprotein (LDL), cholesterol levels were measured. The HFD group showed higher levels of serum TC, TG, and LDL cholesterol than the NC group (*p* < 0.001). The PF group had significantly lower TC (184.60 ± 3.21 mg/dL vs. 224.00 ± 15.90 mg/dL, respectively; *p* < 0.05, Figure 3A), lower TG (104.60 ± 5.54 mg/dL vs. 150.33 ± 11.71 mg/dL, respectively, *p* < 0.01, Figure 3B) and lower LDL cholesterol (36.20 ± 1.52 mg/dL vs. 49.00 ± 4.97 mg/dL, respectively; *p* < 0.05) levels compared to the HFD group (Figure 3C). Additionally, the oral fat tolerance test (OFTT) was performed to evaluate the effect of PF on lipid metabolism. During the OFTT, the TG levels of all groups were highest at 120 min and then gradually decreased. The HFD group showed a higher TG level compared to the NC group throughout the test, and the PF group showed a lower TG level at 120 min (*p* < 0.05) and 180 min (*p* < 0.05) compared to the HFD group (Figure 3D). The HFD group was also significantly higher in the TG AUC compared to the NC group (*p* < 0.05), and the PF group had a lower TG AUC than the HFD group as well (117,618.00 ± 6167.01 vs. 139,920.00 ± 7367.54, respectively; *p* < 0.05, Figure 3E).

### 2.5. Effects of PF on Adipose Tissue Macrophages (ATMs) and Liver Kupffer Cells

**Figure 4:**
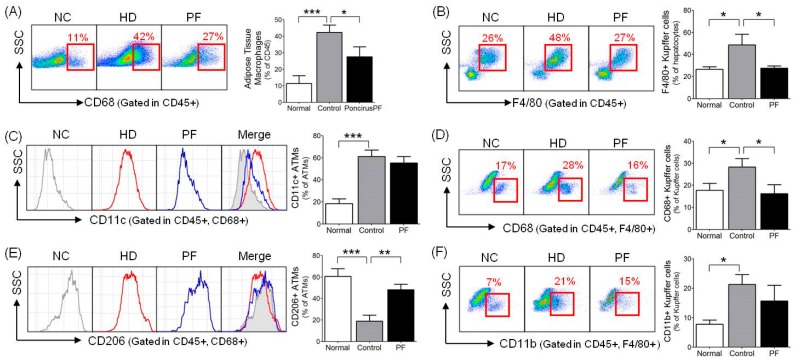
Effect of PF on adipose tissue macrophage (ATM) infiltration rate in (**A**) CD68^+^, (**C**) CD11c^+^, and (**E**) CD206^+^ cells and Kupffer cell infiltration rate in (**B**) F4/80^+^, (**D**) CD68^+^, and (**F**) CD11b^+^ cells. *n* = 6 in each group. Data shown are the mean ± SEM. * *p* < 0.05, ** *p* < 0.01, *** *p* < 0.001, compared with HFD.

The HFD group showed a significantly higher ATM percentage compared to the NC group (*p* < 0.001), and the PF group had a significantly lower ATM percentage compared to the HFD group (27.54 ± 6.08% vs. 42.32 ± 4.60, respectively; *p* < 0.005, Figure 4A). In the case of ATM subpopulations, the proportion of inflammatory CD11c^+^ ATM cells in the total ATM was significantly higher in the HFD group than in the NC group (61.33 ± 5.72% vs. 18.39 ± 4.31%, respectively; *p* < 0.001) while no such significant gap was seen between the PF and the HFD groups (55.44 ± 5.82% vs. 61.33 ± 5.72%, respectively; Figure 4C). Regarding the proportion of anti-inflammatory CD206^+^ ATMs, the total ATM was significantly lower in the HFD group compared to the NC group (*p* < 0.001) and was significantly higher in the PF group compared to the HFD group (48.33 ± 4.94% vs. 18.92 ± 5.69%, respectively; *p* < 0.01, Figure 4E).

To study the effect of PF on liver immune cell composition, the percentage of Kupffer cells among the total mononuclear cells and the percentages of CD68^+^ and CD11b^+^ Kupffer cells were measured. The Kupffer cell percentage was significantly higher in the HFD group compared to the NC group (*p* < 0.05) and was lower in the PF group compared to the HFD group (27.56 ± 2.05% vs. 48.77 ± 9.74%, respectively; *p* < 0.05, Figure 4B). The ratio of phagocytic CD68^+^ Kupffer cells was also significantly higher in the HFD group than in the NC group (*p* < 0.05) and was significantly lower in the PF group compared to the HFD group (16.26 ± 4.07 vs. 28.33 ± 3.84, respectively; *p* < 0.05, Figure 4D). The ratio of cytokine-producing CD11b+ Kupffer cells was significantly higher in the HFD group than in the NC group (*p* < 0.05). The PF group showed a lower percentage of CD11b^+^ Kupffer cells compared to the HFD group, but the results were not significant (15.73 ± 5.30% vs. 21.33 ± 3.35%, respectively; Figure 4F).

### 2.6. Effects of PF on Inflammatory Gene Expression

**Figure 5:**
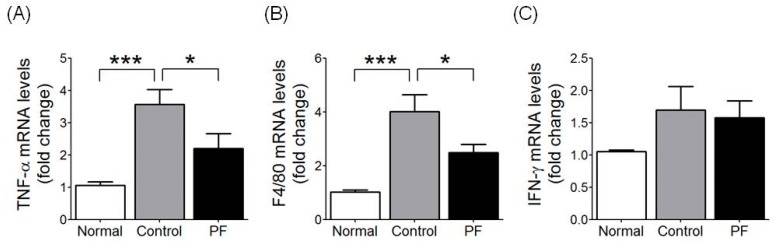
Effect of PF on inflammatory cytokine gene expression in liver. (**A**) TNF-α, (**B**) F4/80 and (**C**) IFN-γ. *n* = 6 in each group. Data shown are the mean ± SEM. * *p* < 0.05, *** *p* < 0.001, compared with HFD.

To evaluate the action of PF on inflammatory cytokines, TNF-α, IFN-γ, and F4/80 mRNA expression was measured in liver tissue. The TNF-α mRNA level was significantly increased in the HFD group compared to the NC group (*p* < 0.001) and was significantly decreased in the PF group compared to the HFD group (*p* < 0.05, Figure 5A). The F4/80 mRNA expression was significantly higher in the HFD group than in the NC group (*p* < 0.001) and was significantly lower in the PF group compared to the HFD group (*p* < 0.05, Figure 5B); however, the three groups shared similar results regarding IFN-γ expression (Figure 5C).

## 3. Discussion

More than ever before, worldwide prevalence of obesity has been an increasing and influencing factor threatening health [19]. Obesity contributes to the development of various metabolic diseases via altered glucose and lipid homeostasis as well as systemic inflammation [20]. While macrophages are the key immune cells involved in the early inflammatory response, obesity patients have increased numbers of ATMs and Kupffer cells. Activated macrophages accelerate systemic inflammation by secreting their own pro-inflammatory mediators, which leads to a disturbance in the insulin signaling pathway [21]. Therefore, regulating inflammatory reactions in adipose tissues and liver to ameliorate insulin resistance could be critical in the treatment of metabolic complications [22]. In this study, PF significantly reduced fat accumulation in liver, enhanced glucose and lipid metabolism, and modulated inflammatory macrophage infiltration as well as cytokine expression in adipose tissue and liver. PF contains high amounts of flavonoids, such as poncirin (over 2%), naringin (over 0.7%), narirutin, hesperidin, neohesperidin, and exhibits anti-inflammatory and anti-obesity properties in vivo and vitro study [15]. Naringin (4’,5,7-trihydroxy flavonone-7-rhamnoglucoside), one of the major PF compounds, was found to ameliorate insulin resistance, dyslipidemia, hepatic steatosis, and kidney damage in a type 2 diabetic rat model by partly regulating oxidative stress, inflammation, and dysregulated adipocytokines production [23]. After treatment with 0.2 g/kg naringin for 10 weeks, reduction in the body weight, liver weight, Lee’s index, and visceral fat were observed in high diet fed C57BL/6 mice [24]. Furthermore, naringin at dosage of 50 and 100 mg/kg for 28 days corrected impaired glucose utilization and insulin insensitivity in diabetic rats [23]. Poncirin, flavanone glycoside, inhibited adipocyte differentiation in mesenchymal stem cells [25]. Further, poncirin exerts an anti-inflammatory effect by inhibiting the LPS-induced expression of inducible nitric oxide synthase, cyclooxygenase 2, TNF-α, and IL-6 through the suppression of NF-kB binding activity in RAW 264.7 macrophages [26]. 

In this experiment, the PF group did not show a significant decrease in weight gained from the HFD and in the epididymal fat pad weight compared to the HFD group. Since the rodent’s epididymal adipose tissue weight is proportional to body weight [27], there was no significant epididymal fat pad loss in the PF group. It is thought that the experiment duration may not have been long enough for weight loss. However, liver weight and liver fat area showed a significant decrease in the PF group compared to the HFD group. High caloric diet impacts fat accumulation in the liver by intra- and extra-hepatic alteration of fat metabolism via directly increasing de novo lipogenesis and indirectly increasing free fatty acid (FFA) influx to the liver by adipose tissue lipolysis [28,29]. Hence, increased liver fat accumulation can be an indirect indicator of obesity and insulin resistance [30]. From the results of liver weight reduction in the PF group, positive effects on insulin resistance can be expected. Several studies have reported the effects of PF extract on anti-obesity. Shim et al. reported that an aqueous extract of PF (200 mg/2 mL/animal/day) suppressed body weight gain by 6% in Sprague-Dawley rats after 10 weeks, likely due to increased rate of intestinal transit time [31], whereas Jia S et al. proved PF extract reduced body weight by 9.21% and modulated glucose and lipid metabolism and hypoglycemic effects on C57BL/6 mice fed a high-fat diet for 13 weeks [17].

In this study, HOMA-IR and OGTT were measured to evaluate the effect of *Poncirus* on the improvement of insulin resistance. The PF group showed significantly lower HOMA-IR compared to the HFD group. HOMA-IR is a useful indicator for evaluating insulin resistance, which is calculated by FBS and fasting insulin level [32]. In the insulin resistance background, a major feature is abnormal glucose and lipid metabolism caused by an abnormal response to insulin, impaired muscle glucose uptake, muscle and liver glycogen synthesis, and overt hyperglycemia [33]. FFA flux is high, TG synthesis and storage are increased, and excess TG is secreted in the form of VLDL in the liver [34]. The excessively produced lipoprotein (VLDL, IDL, LDL) and decreased LPL due to insulin resistance [35] have high TG content and are not efficiently absorbed by the liver, thus creating small dense LDLs, which are the main constituents for atherosclerosis [36]. As such, the treatment of dyslipidemia is also essential in the treatment of metabolic complications [34]. The *Poncirus* group showed significantly lower glucose levels at the 30 min point during OGTT compared to the HFD group. The first 30 min of OGTT is deeply related to the early stages of insulin reaction, and is called the “first phase insulin release”, which is an indicator as to whether insulin resistance or type 2 diabetes will develop [37]. In the OFTT conducted here, the PF group showed a significantly lower level of triglyceride concentration at the 120 and 180 min time points. The concentration of total cholesterol, TG, and LDL-cholesterol were also significantly lower than in the HFD group, showing the positive effects of PF on dyslipidemia. 

Apart from increasing in numbers, adipose tissue macrophages (ATMs) are also phenotypically changed during obesity-induced inflammation [38]. Upon cytokine polarization, macrophages are divided into classically activated macrophages (M1) and alternatively activated macrophages (M2), which present different activators, markers, and functions. While the M1 phenotype stimulates the generation of inflammatory cytokines such as TNF-α, iNOS, CCR2, and IL-12, the M2 phenotype promotes anti-inflammatory cytokines like IL-4 and IL-13, which relieve inflammatory reactions and inhibit the development of insulin resistance [39]. In HFD-induced inflammation mice, the M1/M2 ratio of the macrophages increases by more than 4 times [39,40]. Cell surface markers of M1 ATMs include CD11c, CD40, CD86, HLA-DR, and TLR4 [38,41]. CD11c is predominantly in the pro-inflammatory state [42]. Additionally, cell surface markers of M2 ATMs include CD163, CD204, and CD206 [38,41]. In the analysis of ATM subpopulations, the proportion of CD206^+^ ATMs (M2 phenotype) was significantly higher in the *Poncirus* group compared to the HFD group. Kupffer cells, which are liver macrophages with the F4/80 surface marker in mice [43], are mainly classified into two subsets, CD11b^−^CD68^+^ and CD11b^+^CD68^−^, based on their markers and function [44]. CD68^+^ Kupffer cells have high phagocytic and bactericidal activity, while CD11b^+^ Kupffer cells are involved in the inflammatory response due to their high cytokine-producing capacity [44]. In the study of PF on Kupffer cells, PF significantly lowered the proportion of total Kupffer cells and phagocytic CD68^+^ Kupffer cells. The proportion of cytokine-producing CD11b^+^ Kupffer cells decreased but not significantly, indicating that PF could regulate the expression of phagocytic CD68^+^ cells and inhibit inflammatory conditions. 

In inflammatory cytokine gene expression in Kupffer cells, the HFD group had significantly increased TNF-α and F4/80 mRNA expression levels compared to the NC group, and the PF group was significantly lower in TNF-α and F4/80 mRNA expression compared to the HFD group. 

Once infiltrated into obese adipose tissue, macrophages interact with adipocytes in a paracrine manner through TNF-α production [45], thus increasing lipolysis and activating the TLR4/NF-kB pathway [8,11]. This leads to inflammatory cytokine production, which causes a disturbance in the insulin signaling pathway via inhibiting tyrosine phosphorylation of insulin receptor substrates [46]. 

In this study, we found that PF has favorable effects on hyperglycemia, glucose tolerance, hyperinsulinemia, dyslipidemia, and histopathological fat accumulation in the epididymal fat pad and liver. These results suggest that PF could improve insulin resistance through the inhibition of macrophage-mediated inflammation and enhance glucose and lipid metabolism. Therefore, we conclude that PF is a promising therapeutic agent for insulin resistance and metabolic complications, and additional further research including clinical trials will be necessary to confirm these results.

## 4. Materials and Methods

### 4.1. Preparation of Poncirus fructus

*Poncirus fructus* was obtained from the Department of Pharmaceutical Preparation of Korean Medicine, Korean Medical Hospital, Kyung Hee University, Seoul, Korea. We tested the drug quality according to the standards of the Korea Food & Drug Administration and those of our hospital. Dried PF (1000 g) was added to ethanol (1500 mL, 80%) and boiled for 2 h at 100 °C. The sieve-filtrated solvents were concentrated with a rotary evaporator (Model NE-1, EYELA Co., Tokyo, Japan) and dried with a freeze dryer (Model FD-1, EYELA Co., Tokyo, Japan). The extracts were added to distilled water (1 g/10 mL) and boiled for 2 h at 95 °C. The boiled solution was centrifuged at 14,000 rpm for 20 min, and supernatant was obtained. The final extract weight of PF was 160 g.

### 4.2. Experimental Design and Animals

Six-week-old male C57BL/6 mice weighing 19 ± 2 g were purchased from Central Lab Animal Inc. (Seoul, Korea) and were housed in stainless steel cages in an air-conditioned room controlled at 22 ± 1 °C and at 40% to 70% relative humidity under a 12:12 h dark/light schedule. Animals freely received diet and water for one week. After adapting to the lighting conditions for that one week, the mice were randomly assigned to one of three groups: Normal chow (NC, *n* = 6), high-fat diet (HFD, *n* = 6), and PF (*n* = 6). Except for those in the NC group, all mice were fed an HFD (60% energy by fat, % kcal; carbohydrate/protein/fat = 20:20:60), which is known to induce obesity, for 17 weeks. After confirmation that 8 weeks of HFD feeding made a significant difference (*p* < 0.001) in body weight between the NC group and the other groups, PF (500 mg/kg body weight, dissolved in distilled water) was orally administered daily for 9 weeks, while the NC and HFD group received normal saline. The PF dose was selected based on the yield of PF extract (16%) and the content of naringin (0.7%) which is a major flavonoid of PF, and showed anti-obesity and anti-inflammatory effects in the previous study [23]. 

All experiments were carried out according to the principles outlined in the NIH Guide for the care and use of laboratory animals and study protocol was approved at 13-04-2018 by the Animal Care Committee of the Animal Center at Kyung Hee Medical Center (KHMC-IACUC201813). 

### 4.3. Oral Glucose Tolerance Test and Blood Analysis

Oral glucose tolerance tests (OGTTs) were carried out at week 14. Glucose was added to distilled water and administered to each mouse (2 g glucose per kg body weight) through a stomach tube after 14 h of fasting. Blood glucose was measured at 0, 30, 60, 120, and 180 min time points after administration using a strip-operated blood glucose sensor (Accu-Chek Performa, Australia). Blood was collected from the tail vein of each mouse. Glucose area under the curve (AUC) in the OGTT was calculated from measurements taken before (0 min) and after (up to 180 min) glucose administration using the trapezoidal rule, which is a numerical integration method used to approximate the integral or the AUC.

At week 15, blood was obtained from the tail vein of each mouse after 6 h of fasting to measure insulin concentration. Serum insulin concentration was measured using an ultrasensitive mouse insulin ELISA kit (Crystal Chem Inc., Elk Grove Village, IL, USA). From fasting blood glucose and insulin concentration, insulin resistance was assessed using the homeostatic model assessment of insulin resistance (HOMA-IR). At the end of the experiment, after 14 h of fasting, serum total cholesterol (TC), low density lipoprotein (LDL) cholesterol, and TG levels were measured.

### 4.4. Oral Fat Tolerance Test (OFTT)

At week 15, fasting triglyceride (TG) levels (0 min) were measured from tail vein blood after 14 h of fasting. Then, olive oil (2 mL/kg body weight) was administered orally, and TG measurements were taken from tail vein blood samples taken at 120, 180, 240, and 360 min time points after fat administration. The TG measurements were performed using an Accutrend Plus meter (Roche, Brighton, MA, USA). The TG AUC in the OFTT was calculated from measurements taken before (0 min) and after (up to 360 min) fat administration.

### 4.5. RNA Isolation and Analysis of Gene Expression

At week 17, the mice were sacrificed, and livers were dissected, and total RNA was extracted from livers using a Mini RNA Isolation II^TM^ kit (ZYMO RESEARCH, Irvine, CA, USA) according to the manufacturer’s instructions. To evaluate gene expression including TNF-α, interferon gamma (IFN-γ), and F4/80, quantitative real time-polymerase chain reaction (qRT-PCR) assays were performed. Prior to qRT-PCR, the complementary DNA (cDNA) was synthesized using an Advantage RT for PCR Kit (Clontech, Mountain View, CA, USA). To the cDNA obtained through reverse transcription PCR, 2X SYBR Reaction buffer, primers, and dH_2_O were added, and qRT-PCR was carried out using a 7900HT Fast Real-Time PCR System (Applied Biosystems^®^, Waltham, MA, USA). For gene expression analysis, the threshold cycle for each gene, obtained with SDS Software 2.4 (Applied Biosystems^®^, Waltham, MA, USA), was converted to relative quantitation based on GAPDH, and the fold change was calculated. The fold change value of each experimental group was normalized according to the NC group, which was defined as 1.

### 4.6. Isolation of Stromal Vascular Cells (SVCs) and Liver Immune Cells

At week 17, harvested epididymal fat pads were put into a solution composed of phosphate-buffered saline (PBS, Gibco, Waltham, MA, USA) and 2% bovine serum albumin (BSA, Gibco, Waltham, MA, USA) and minced into 1 to 2 mm pieces with round-shaped scissors. After adding collagenase (Sigma, St. Louis, MO, USA) and DNase I (Roche, Brighton, MA, USA), a 100 μm cell strainer (BD Biosciences, San Jose, CA, USA) was used to remove extraneous tissue. The liver was perfused with PBS (pH 7.0) through a needle inserted into the portal vein, and then liver tissue was placed in a 60 mm petri dish with RPMI 1640 medium containing 100 mL/L fetal calf serum (FCS) and pulverized into small pieces. The sample was filtered through a 200-gauge stainless mesh and mixed with 9 mL of PBS, 8 mL of Percoll (final 36.3%), and 200 μL heparin, and the mixture was centrifuged at 2000 rpm for 20 min. After removal of the supernatant containing parenchymal cells, 1X ACK lysis buffer (Lonza, Houston, TX, USA) was added to the pellet to dissolve the red blood cells. The sample was finally centrifuged at 1500 rpm for 5 min to obtain non-parenchymal cells containing immune cells collected in the lower layer. 

### 4.7. Fluorescence-Activated Cell Sorting (FACS) Analysis of Adipose Tissue Macrophages (ATMs) and Kupffer Cells

Each sample was prepared to contain 10^6^ cells. A mixture of FcBlock reagent (BD Pharmingen, San Jose, CA, USA) and fluorophore-conjugated antibodies was added to each sample. The antibodies used for the analysis of ATMs were CD45-APC Cy7 (BioLegend, San Diego, CA, USA), CD68-APC (BioLegend), CD11c-phycoerythrin (CD11c-PE, BioLegend), and CD206-FITC (BioLegend). Antibodies for liver Kupffer cell analysis included CD45-FITC (BioLegend), F4/80-APC (BioLegend), CD68-PE (BioLegend), and CD11b-PerCp CY5.5 (BioLegend). After washing with 2% FBS/PBS solution, each sample was centrifuged at 1500 rpm and transferred in a fluorescence-activated cell sorting (FACS) tube. The analysis was conducted using a FACS Calibur flow cytometer (BD Bioscience, USA). The percentage of ATMs with CD45^+^ CD68^+^, CD45^+^ CD68^+^ CD206^+^, and CD45^+^ CD68^+^ CD11c^+^ expressed and the percentage of Kupffer cells with CD45^+^ F4/80^+^, CD45^+^ F4/80^+^ CD68^+^, and CD45^+^ F4/80^+^ CD11b^+^ expressed were analyzed using FlowJo (Tree Star, Inc., Ashland, OR, USA). 

### 4.8. Histological Analyses of Adipose Tissue and Liver

Obtained epididymal fat pad and liver samples were fixed in 10% neutral buffered formalin and embedded in paraffin to make paraffin blocks. Each block was sliced into 4 μm-thick sections with a microtome and attached to a gelatin coated slide. Two sections per animal were stained with hematoxylin and eosin, and digital images were obtained using a high-resolution camera-mounted optical microscope (Olympus BX-50, Olympus Optical, Tokyo, Japan) connected to a computer. Using ImageJ, the adipocyte size in fat tissue and the fat area in liver tissue were measured.

### 4.9. Statistical Analysis

Statistical significances were examined by one-way analysis of variance (ANOVA) followed by Tukey’s post hoc test using the GraphPad PRISM statistical package (version 4.03, GraphPad Software Inc., San Diego, CA, USA). The data are presented as the mean ± standard error. *P* values were determined at *p* < 0.05.

## References

[1] Fantuzzi G. (2005). Adipose tissue, adipokines, and inflammation. J. Allergy Clin. Immunol..

[2] Ellulu M.S., Patimah I., Khaza’ai H., Rahmat A., Abed Y. (2017). Obesity and inflammation: The linking mechanism and the complications. Arch. Med. Sci..

[3] Lumeng C.N., Saltiel A.R. (2011). Inflammatory links between obesity and metabolic disease. J. Clin. Invest..

[4] Kershaw E.E., Flier J.S. (2004). Adipose tissue as an endocrine organ. J. Clin. Endocrinol. Metab..

[5] Hosogai N., Fukuhara A., Oshima K., Miyata Y., Tanaka S., Segawa K., Furukawa S., Tochino Y., Komuro R., Matsuda M. (2007). Adipose tissue hypoxia in obesity and its impact on adipocytokine dysregulation. Diabetes.

[6] Gregor M.F., Hotamisligil G.S. (2007). Thematic review series: Adipocyte biology. Adipocyte stress: The endoplasmic reticulum and metabolic disease. J. Lipid Res..

[7] Surmi B.K., Hasty A.H. (2008). Macrophage infiltration into adipose tissue: Initiation, propagation and remodeling. Future Lipidol..

[8] Cawthorn W.P., Sethi J.K. (2008). Tnf-alpha and adipocyte biology. FEBS Lett..

[9] Trayhurn P., Wood I.S. (2004). Adipokines: Inflammation and the pleiotropic role of white adipose tissue. Br. J. Nutr..

[10] Shi H., Kokoeva M.V., Inouye K., Tzameli I., Yin H., Flier J.S. (2006). Tlr4 links innate immunity and fatty acid-induced insulin resistance. J. Clin. Invest..

[11] Guilherme A., Virbasius J.V., Puri V., Czech M.P. (2008). Adipocyte dysfunctions linking obesity to insulin resistance and type 2 diabetes. Nat. Rev. Mol. Cell Biol..

[12] Hubler M.J., Kennedy A.J. (2016). Role of lipids in the metabolism and activation of immune cells. J. Nutr. Biochem..

[13] Lee H.T., Seo E.K., Chung S.J., Shim C.K. (2005). Prokinetic activity of an aqueous extract from dried immature fruit of poncirus trifoliata (l.) raf. J. Ethnopharmacol..

[14] Yu D.-J., Jun J.-H., Kim T.-J., Suh D.-K., Youn D.-H., Kim T.-W. (2015). The relaxing effect of poncirus fructus and its flavonoid content on porcine coronary artery. Lab. Anim. Res..

[15] Jang Y., Kim E.K., Shim W.S. (2018). Phytotherapeutic effects of the fruits of poncirus trifoliata (l.) raf. On cancer, inflammation, and digestive dysfunction. Phytother. Res..

[16] Guo H.F., Saravanakumar K., Wang M.H., Kim H.Y. (2019). Antioxidant activity of ethanol extract of poncirus fructus immaturus. Res. J. Biotechnol..

[17] Jia S., Gao Z., Yan S., Chen Y., Sun C., Li X., Chen K. (2016). Anti-obesity and hypoglycemic effects of poncirus trifoliata l. Extracts in high-fat diet c57bl/6 mice. Molecules.

[18] Lee H.-T., Seo E.-K., Chung S.-J., Shim C.-K. (2005). Effect of an aqueous extract of dried immature fruit of poncirus trifoliata (l.) raf. On intestinal transit in rodents with experimental gastrointestinal motility dysfunctions. J. Ethnopharmacol..

[19] Hruby A., Hu F.B. (2015). The epidemiology of obesity: A big picture. PharmacoEconomics.

[20] Parhofer K.G. (2015). Interaction between glucose and lipid metabolism: More than diabetic dyslipidemia. Diabetes Metab. J..

[21] Weisberg S.P., McCann D., Desai M., Rosenbaum M., Leibel R.L., Ferrante A.W. (2003). Obesity is associated with macrophage accumulation in adipose tissue. J. Clin. Invest..

[22] Xu H., Barnes G.T., Yang Q., Tan G., Yang D., Chou C.J., Sole J., Nichols A., Ross J.S., Tartaglia L.A. (2003). Chronic inflammation in fat plays a crucial role in the development of obesity-related insulin resistance. J. Clin. Invest..

[23] Sharma A.K., Bharti S., Ojha S., Bhatia J., Kumar N., Ray R., Kumari S., Arya D.S. (2011). Up-regulation of ppargamma, heat shock protein-27 and -72 by naringin attenuates insulin resistance, beta-cell dysfunction, hepatic steatosis and kidney damage in a rat model of type 2 diabetes. Br. J. Nutr..

[24] Pu P., Gao D.-M., Mohamed S., Chen J., Zhang J., Zhou X.-Y., Zhou N.-J., Xie J., Jiang H. (2012). Naringin ameliorates metabolic syndrome by activating amp-activated protein kinase in mice fed a high-fat diet. Arch. Biochem. Biophys..

[25] Yoon H.Y., Yun S.I., Kim B.Y., Jin Q., Woo E.R., Jeong S.Y., Chung Y.S. (2011). Poncirin promotes osteoblast differentiation but inhibits adipocyte differentiation in mesenchymal stem cells. Eur. J. Pharmacol..

[26] Kim J.B., Han A.R., Park E.Y., Kim J.Y., Cho W., Lee J., Seo E.K., Lee K.T. (2007). Inhibition of lps-induced inos, cox-2 and cytokines expression by poncirin through the nf-kappab inactivation in raw 264.7 macrophage cells. Biol. Pharm. Bull..

[27] Suzuki M., Ding Q., Muranaka S., Kigure M., Kojima M., Terada M., Kataoka N., Hagiwara A., Kromkhun P., Moritani N. (2008). Correlation between body weight (epididymal fat) and permeation rate of serum leptin through the blood-brain barrier (bbb) in male rats aged 8 months. Exp. Anim..

[28] Schonfeld G., Yue P., Lin X., Chen Z. (2008). Fatty liver and insulin resistance: Not always linked. Trans. Am. Clin. Clim. Assoc..

[29] Lambert J.E., Ramos-Roman M.A., Browning J.D., Parks E.J. (2014). Increased de novo lipogenesis is a distinct characteristic of individuals with nonalcoholic fatty liver disease. Gastroenterology.

[30] Perry R.J., Samuel V.T., Petersen K.F., Shulman G.I. (2014). The role of hepatic lipids in hepatic insulin resistance and type 2 diabetes. Nature.

[31] Shim W.S., Back H., Seo E.K., Lee H.T., Shim C.K. (2009). Long-term administration of an aqueous extract of dried, immature fruit of poncirus trifoliata (l.) raf. Suppresses body weight gain in rats. J. Ethnopharmacol..

[32] Bonora E., Targher G., Alberiche M., Bonadonna R.C., Saggiani F., Zenere M.B., Monauni T., Muggeo M. (2000). Homeostasis model assessment closely mirrors the glucose clamp technique in the assessment of insulin sensitivity: Studies in subjects with various degrees of glucose tolerance and insulin sensitivity. Diabetes Care.

[33] Abdul-Ghani M.A., DeFronzo R.A. (2010). Pathogenesis of insulin resistance in skeletal muscle. J. Biomed. Biotechnol..

[34] Ginsberg H.N., Zhang Y.L., Hernandez-Ono A. (2005). Regulation of plasma triglycerides in insulin resistance and diabetes. Arch. Med. Res..

[35] Albalat A., Saera-Vila A., Capilla E., Gutiérrez J., Pérez-Sánchez J., Navarro I. (2007). Insulin regulation of lipoprotein lipase (lpl) activity and expression in gilthead sea bream (sparus aurata). Comp. Biochem. Physiol. Part B Biochem. Mol. Biol..

[36] Sparks J.D., Sparks C.E., Adeli K. (2012). Selective hepatic insulin resistance, vldl overproduction, and hypertriglyceridemia. Arter. Thromb. Vasc. Biol..

[37] Gerich J.E. (2002). Is reduced first-phase insulin release the earliest detectable abnormality in individuals destined to develop type 2 diabetes?. Diabetes.

[38] Dalmas E., Clement K., Guerre-Millo M. (2011). Defining macrophage phenotype and function in adipose tissue. Trends Immunol..

[39] Lumeng C.N., Bodzin J.L., Saltiel A.R. (2007). Obesity induces a phenotypic switch in adipose tissue macrophage polarization. J. Clin. Invest..

[40] Cusi K. (2012). Role of obesity and lipotoxicity in the development of nonalcoholic steatohepatitis: Pathophysiology and clinical implications. Gastroenterology.

[41] Komohara Y., Fujiwara Y., Ohnishi K., Shiraishi D., Takeya M. (2016). Contribution of macrophage polarization to metabolic diseases. J. Atheroscler. Thromb..

[42] Wentworth J.M., Naselli G., Brown W.A., Doyle L., Phipson B., Smyth G.K., Wabitsch M., O’Brien P.E., Harrison L.C. (2010). Pro-inflammatory cd11c+cd206+ adipose tissue macrophages are associated with insulin resistance in human obesity. Diabetes.

[43] Austyn J.M., Gordon S. (1981). F4/80, a monoclonal antibody directed specifically against the mouse macrophage. Eur. J. Immunol..

[44] Kinoshita M., Uchida T., Sato A., Nakashima M., Nakashima H., Shono S., Habu Y., Miyazaki H., Hiroi S., Seki S. (2010). Characterization of two f4/80-positive kupffer cell subsets by their function and phenotype in mice. J. Hepatol..

[45] Suganami T., Nishida J., Ogawa Y. (2005). A paracrine loop between adipocytes and macrophages aggravates inflammatory changes: Role of free fatty acids and tumor necrosis factor alpha. Arter. Thromb. Vasc. Biol..

[46] Chen L., Chen R., Wang H., Liang F. (2015). Mechanisms linking inflammation to insulin resistance. Int. J. Endocrinol..

